# Interdisciplinary Management of Bilateral Congenital Lateral Incisor Agenesis

**DOI:** 10.1155/2023/5576050

**Published:** 2023-07-10

**Authors:** Margaux Vignon, Typhaine Bensaidani, Serge Soliveres, Philippe Bousquet

**Affiliations:** Department of Periodontology and Implantology, Faculty of Dentistry, University of Montpellier, Montpellier, France

## Abstract

Management of lateral incisor agenesis is a real challenge and needs a strong collaboration between surgical, aesthetic, and orthodontic dentistry. This case report managed upper lateral incisor agenesis with an orthodontic treatment leading to open spaces and placement of cuspids in lateral incisor area to avoid implant placement in maxillary anterior region. Temporary rehabilitation phase, using resin injected tray and removable partial denture, has been placed to maintain mesiodistal dimensions and restore aesthetic during pre-implant analysis and osseointegration. Once osseointegration was fully obtained, keratinized tissue augmentation has been obtained using connective tissue graft. Then, temporary implant fixed crowns allowed soft tissue modeling during interim rehabilitation. Finally, screw-retained permanent crowns were placed to fully restore aesthetic and function. This case goal was to optimize final results and reach patient complete satisfaction using orthodontic treatment combined with implant rehabilitation, and coupled with interdisciplinary management and well-time sequencing treatment.

## 1. Introduction

Teeth agenesis are frequent and represent serious public health concern with psychological impact during childhood, adolescence, and sometimes adult life. In our practice, especially concerning maxillary lateral incisors, it is a real aesthetic challenge [1]. Bilateral expression of missing upper laterals represents 62% of this genetic anomaly [2] and causes aesthetic and function failures like diastemata, smile asymmetry, and mesially positioned canines.

Most of the patients receive orthodontic correction with different treatment possibilities. Space can be closed and canine reshaped in lateral incisor, or space can be maintained or opened, and the replacement of the missing incisor made by fixed rehabilitation like bridge or implant-supported crown [3, 4]. An important advantage of opened spaces treatment is to restore canine guidance during lateral movements.

But lateral incisors have a key function in the anterior guide and an important aesthetic impact, making its prosthetic rehabilitation complex, particularly implant-supported ones.

In fact, implants are more and more used to replace congenitally lateral incisors in young adult patients. But this specific area is a real challenge for implantology because of narrow dimensions (alveolar crest and prosthetic space) and adjacent root axis. The placement of implant is, therefore, dependent on orthodontic opened space, and in a large part of the case, ridge augmentation and mucogingival management are necessary [5].

Moreover, anterior alveolar growth, despite its decrease after adolescence, continues all lifelong. Animal and clinical studies have shown that implants do not follow the growth [6, 7]. This immobility can lead to infraocclusion and aesthetic problems, particularly, in young adults but also in older patients [8], with an increased risk for patients with an hyperdivergent growth pattern and for women [9].

Thereby, a well time management and pre-treatment screening are necessary to achieve implant-supported rehabilitation in case of congenitally missing lateral incisors. Orthodontic treatment, by creating sufficient space and optimizing occlusion relationship, helps us to reach ideal reconstruction. This case report is an example of time sequencing interdisciplinary treatment for rehabilitation of bilateral missing lateral incisors with an innovative approach: mesialisation of the canines combined with opened space and implant fixed rehabilitation in canine area with periodontal tissue management.

## 2. Case Presentation

A woman about 30 years old, with no systematic health issues and non-smoker, consulted the Odontology Department of University Hospital Center of Montpellier (Montpellier, France) to manage aesthetic damages due to congenital missing lateral incisors. After being well informed of every alternative treatment, the patient consented to be treated with an orthodontic treatment associated with delayed implant fixed crowns and provided written informed consent.

### 2.1. Orthodontic Treatment

**Figure 1:**
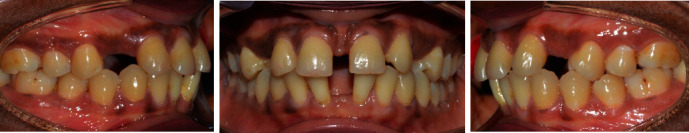
Initial clinical situation showing the main patient complaint: inaesthetic diastemata.

**Figure 2:**
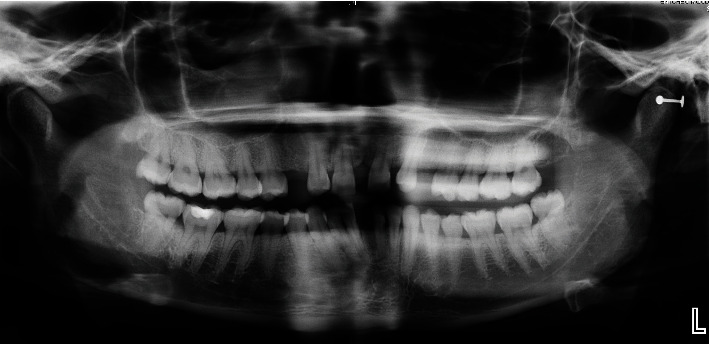
Initial panoramic radiography.

**Figure 3:**
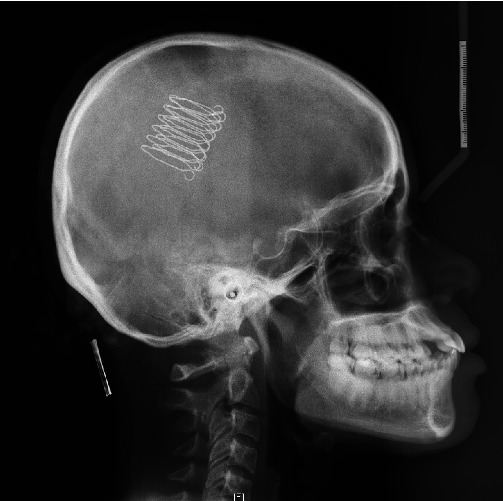
Initial profile teleradiography.

Treatment initially involved mesializing the canines to open up the spaces and reshaping the cuspids with ameloplastia. The choice of opening spaces was required after an accurate clinical radiographic analysis. Indeed, the patient presented several diastemas as well as a mesioposition of canines before orthodontic treatment (Figure 1). In addition, the patient had asymmetry in her wisdom teeth with agenesis of tooth number 16, therefore, excluding the possibility of mesialising molars (Figure 2). Finally, the cephalometric analysis showed a skeletal Class III (angle ANB = 1°) as well as a normodivergence with a tendency to hypodivergence (angle FMA = 23°) (Figure 3). Because of the sagittal skeletal relationships, the solution of opening spaces was selected. Mediation of the canines in place of lateral incisors was performed, and canines were rehabilitated with implants [10]. This solution does not accentuate the negative overjet already present in Class III patients [11]. A 2-year multibracket orthodontic treatment in the vestibule area closed the diastemas by repositioning the incisors with maximum bimaxillary anchorage.

At the end of the orthodontic treatment, prosthetic and bone spaces were sufficient for the placement of 6 and 11 implants. A subtractive coronoplasty was realized to make up the canines as lateral incisors. During this treatment, lingual rehabilitation was performed to perpetuate the results. Ultimately, a retention was performed to maintain the spaces before the implants were placed. At the end of the orthodontic treatment, patient was addressed to the Implantology Department to restore edentulous spaces with implant-supported crowns.

### 2.2. Pre-Surgical Screening

**Figure 4:**
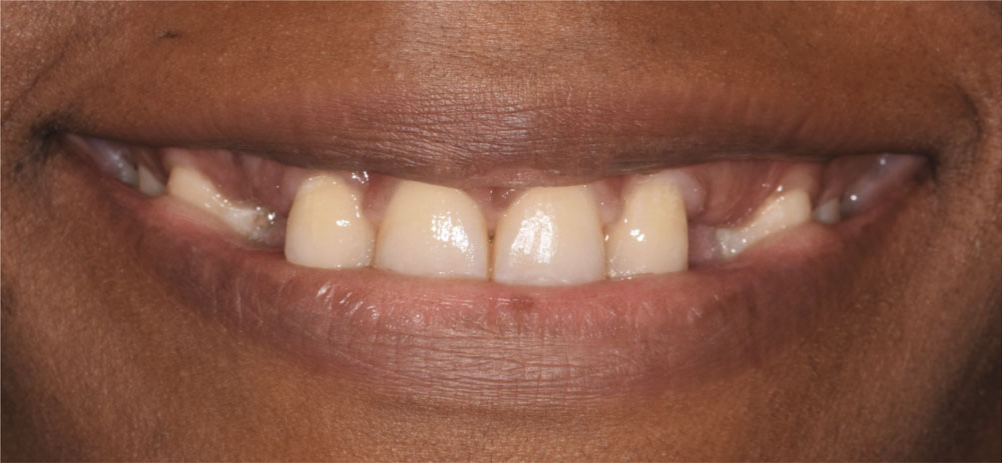
Average smile line during extraoral examination and after orthodontic treatment.

Upon extraoral examination, the patient presented an average smile line with visibility of her interdental papilla and partial edentulous spaces in canine area (Figure 4).

**Figure 5:**
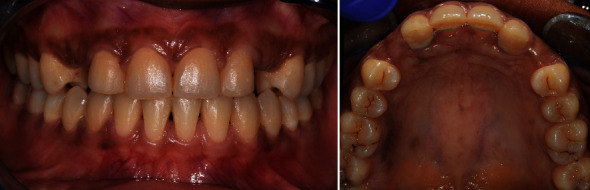
Post-orthodontic maxillary occlusal and frontal views showing maintained restorative spaces with palatal fixed retainer.

On intraoral examination, a healthy periodontal status with a thick maxillary phenotype presenting gingival pigmentations was observed. Restorative spaces were about 7.5 mm and were maintained with fixed retainers and maxillary removable resin injected retainer in canine area (Figure 5).

**Figure 6:**
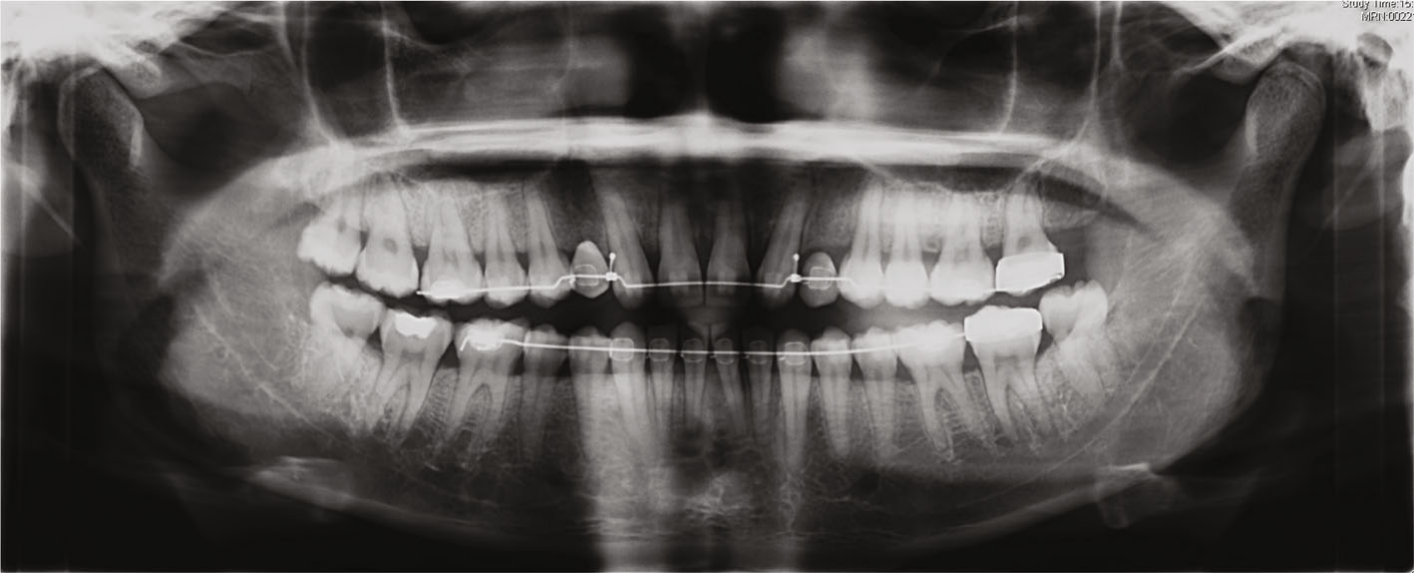
Panoramic radiography at the end of the orthodontic treatment.

Regarding the panoramic radiography realized at the end of the orthodontic treatment, canines' root axes were divergent, reducing mesiodistal bone space. But root axes were parallel to bicuspid ones, making the implant placement easier (Figure 6).

**Figure 7:**
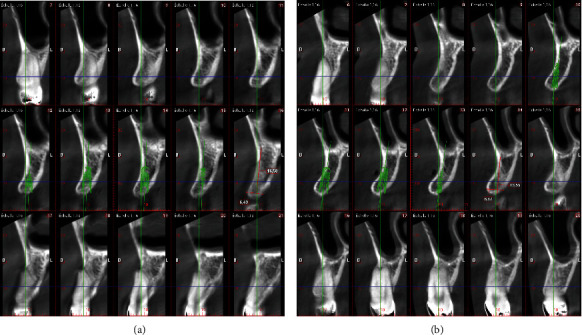
Sagittal views from the computed tomography (a) on the region of tooth number 6 with the implant prefiguration (blue) and (b) on the region of tooth number 11.

A pre-operative computed tomography was performed to analyze ridge dimensions for implant placement surgery. The mesiodistal bone spaces were about 7 mm in canine position 6 and 6 mm in position 11. On both sides, alveolar crest was narrow (7 mm) and presented a buccal undercut (Figure 7).

The patient, with no contraindications to surgery, was informed that a ridge augmentation could be necessary during the surgical phase to avoid vestibular fenestration, and narrow implants have been chosen to fit the ridge width and maintain a minimum of 1.5 mm peripheric bone around implant.

### 2.3. Interim Restoration and Surgeries

**Figure 8:**
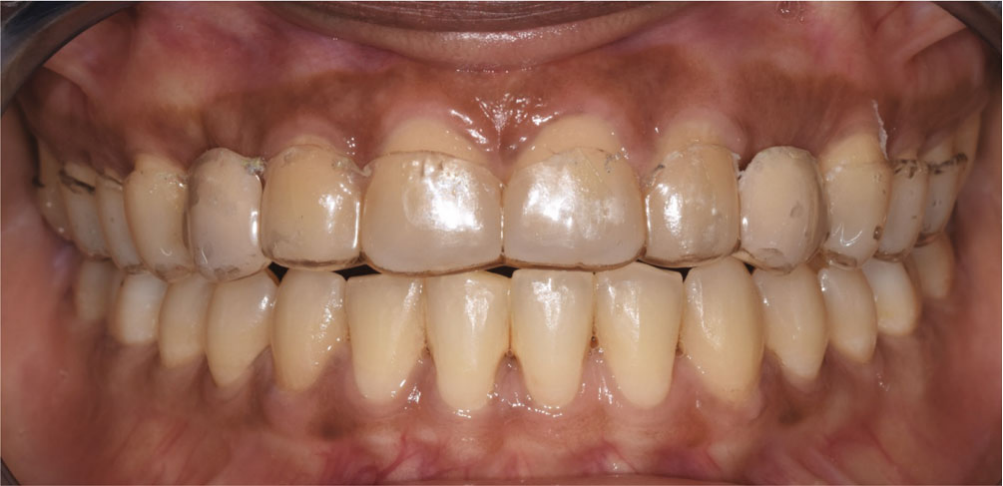
Immediate interim restoration of teeth numbers 6 and 11 using composite injected resin tray.

Impressions of the maxilla and mandible with irreversible hydrocolloid (Alginoplast®, Kulzer Mitsu Chemicals Groups, Hanau, Germany) were practiced, and the diagnostic setup for the final restoration was made in order to prepare a composite injected resin tray as interim restoration (Figure 8). Careful attention was taken to maintain space between gingiva and tray, in order to avoid healing interactions.

**Figure 9:**
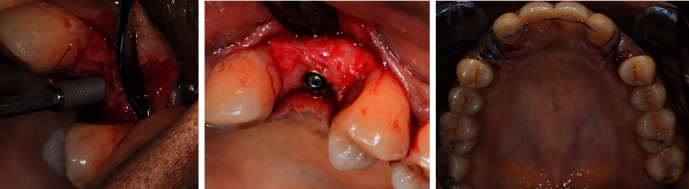
Pre- and post-operative illustrations: implant placement on the region of tooth number 11, implant platform at bone level with peripheric one, primary closure with resorbable sutures.

During surgery, full thickness flap was raised, and two implants were placed with an insertion torque of more than 30 N cm (3.1 × 10 mm; Eztetic®, Zimmer Biomet, Palm Beach Gardens, FL, USA) on the region of teeth numbers 6 and 11. Because of the underestimated alveolar ridge dimension visualized on the computed tomography, guided bone regenerations were not indicated and vestibular fenestration have been avoided. Surgical healing screw (CCNSP, Zimmer Biomet, Palm Beach Gardens, FL, USA) was placed on each implant, and operative sites were primarily closed using resorbable sutures 4.0 (Vicryl®, Ethicon) (Figure 9).

Resin tray immediate interim rehabilitation was placed to ensure patient's cosmetic satisfaction and maintain edentulous space during early healing process (D + 15). After that, a removable partial denture was placed for 3 months, until osseointegration.

Mucogingival management during second stage implant surgery was performed after osseointegration, and healing collars were placed at the same time (CHCNP33, Zimmer Biomet, Palm Beach Gardens, FL, USA).

**Figure 10:**
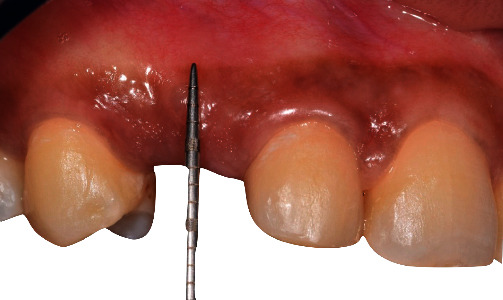
Pre-operative clinical situation showing a lack of keratinized tissue around implant in the region of teeth number 6 with a pigmented gingiva.

**Figure 11:**
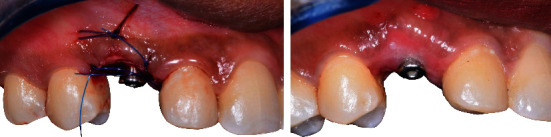
Connective tissue graft during second stage implant surgery at D0 and D + 15.

Because of a lack of keratinized tissue (KT) around implant number 6, a connective tissue graft using envelope flap technique was performed in this site using crestal harvesting to avoid changing gingival pigmentations (Figures 10 and 11).

**Figure 12:**
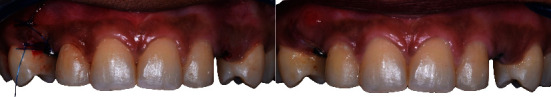
Frontal view of second stage implant surgery: D0 and D + 15.

**Figure 13:**
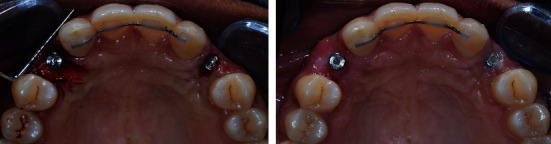
Occlusal maxillary view of second stage implant surgery: D0 and D + 21.

In implant site number 11, a simple incision allowed the placement of the healing collar. The two surgeries were practiced at the same time, and control visits were realized at 2 weeks and 3 weeks (Figures 11, 12, and 13).

### 2.4. Temporary Implant Fixed Crowns

At D + 21 after second stage implant surgery, the maxilla was impressed using open-tray procedure and dual-mix technique with silicones of different viscosities. First, healing collars were removed, and direct transfers were placed using retaining screw (CHCNP33, Zimmer Biomet, Palm Beach Gardens, FL, USA). Then, light silicone was charged around transfers, and a tray filled with heavy silicone was inserted in the upper jaw. After impression time, transfers were unscrewed and trial disinserted. Finally, implant analogs (CIANP, Zimmer Biomet, Palm Beach Gardens, FL, USA) were screwed to direct transfers into the impression. The mandible impression was classically realized with irreversible hydrocolloid (Alginoplast®, Kulzer Mitsu Chemicals Groups, Hanau, Germany). Occlusion cire and teeth color were finally recorded, and information were sent to the laboratory.

**Figure 14:**
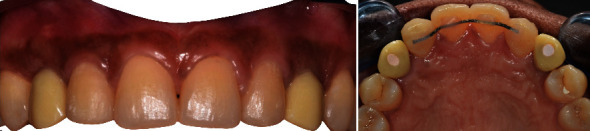
Frontal and occlusal views of the integration of temporary implant-supported crowns.

**Figure 15:**
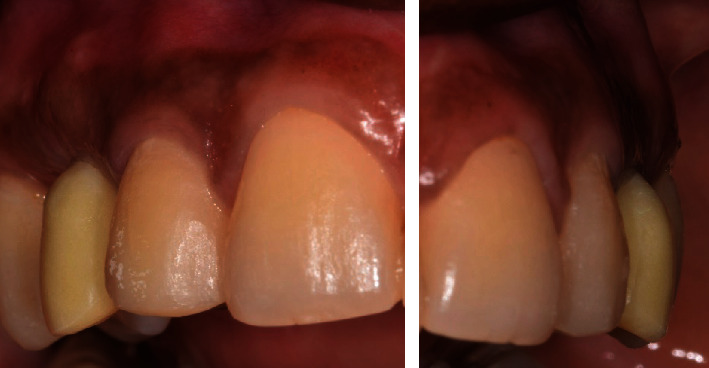
Management of peri-implant profiles in positions 6 and 11.

Temporary implant directly fixed crows were tried, and occlusion was adjusted in order to obtain canine guidance in lateral movement but no interdental contact in static occlusion (Figure 14). With these temporary crowns, patient had function and aesthetic rehabilitation, and profile emergence was modeled with resin adjunction for the future definitive crowns (Figure 15).

### 2.5. Teeth Whitening

After orthodontic treatment, the patient was complaining about teeth color, with a “yellow” aspect [12]. A step of at-home tooth bleaching was performed between temporary and definitive restoration and using 10% carbamide peroxide with adaptable trays (Polanight®, SDI).

**Figure 16:**
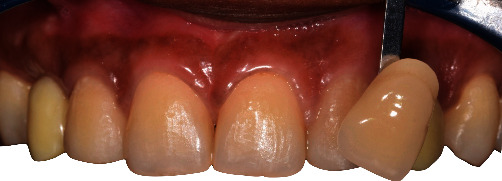
Initial tint before tooth bleaching.

**Figure 17:**
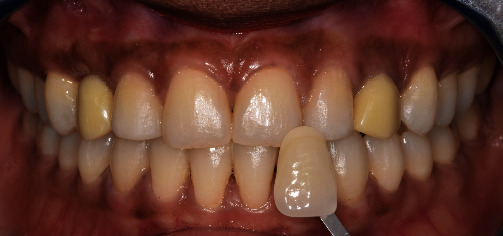
Tint at 1 week after at-home bleaching (2M2) showing the difference between natural denture and temporary crowns in positions 6 and 11.

Teeth color was visually measured by the patient and clinicians: the initial teeth color was 3M2 (Figure 16) and after 7 days of overnight at-home bleaching, the final teeth color was 2M2 (Vita 3D-Master®, Vita) (Figure 17). Patient was satisfied by the aspect of her teeth, more luminous and less saturated. The bleaching treatment was continued for one more week, and tint stabilization was reached at 1 month, allowing final implant fixed restoration in positions 6 and 11.

### 2.6. Final Prosthetic Rehabilitation

**Figure 18:**
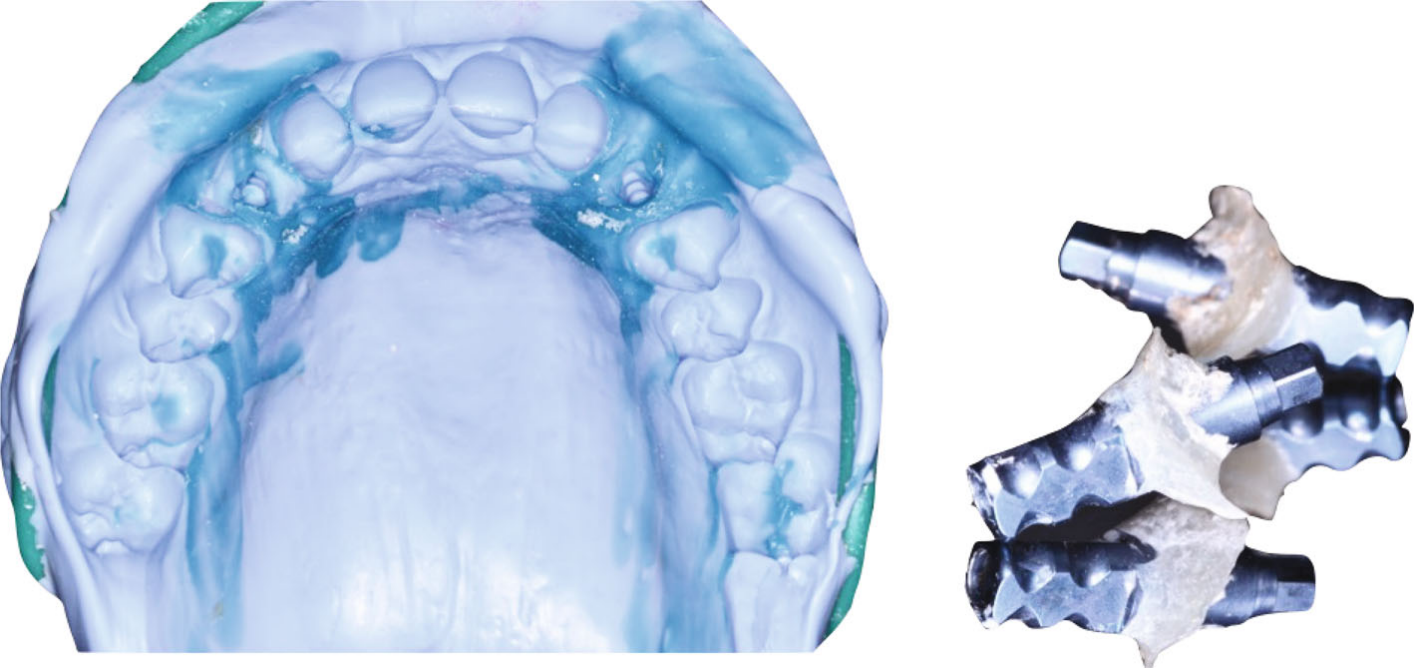
Supraimplant silicone dual-mix impression combined to emergence profile impression with flowable composite.

Once soft tissue has matured and teeth color was stabilized, final implant fixed crowns have been realized. Impression technique was similar to the interim phase except the first step. In fact, a record of the emergence profile was made using flowable resin on the direct transfer (Figure 18).

**Figure 19:**
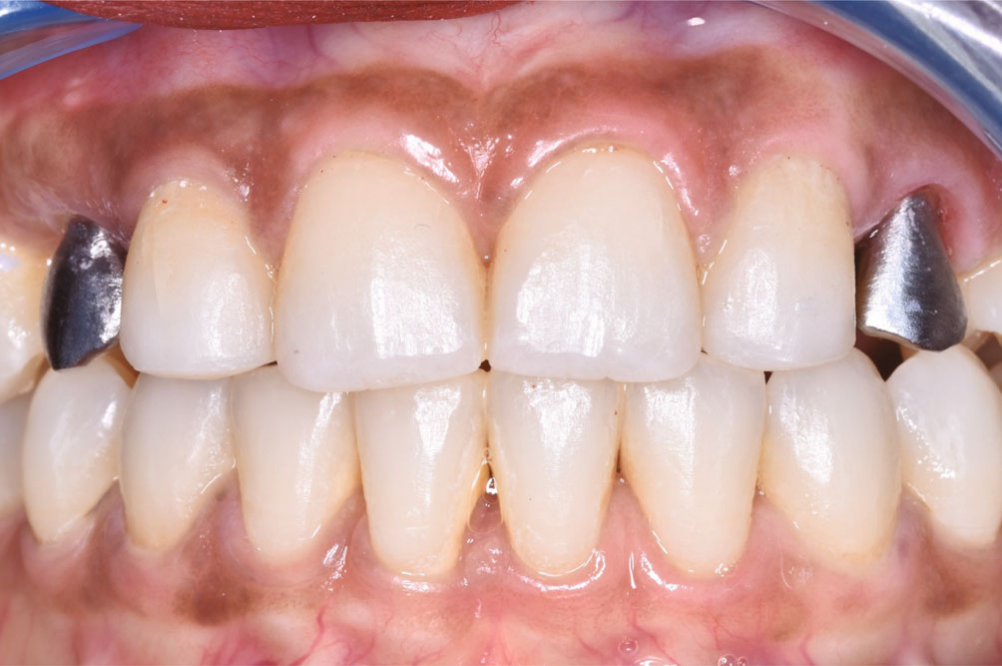
Fitting of the framework showing space for ceramic.

Due to the available prosthetic height, individualized milled direct-implant frameworks were made (Zfx, Germany, company of the ZimVie Group) (Figure 19). They allow better management of the emergence profile and passivity, compared to a conventional cast framework or standardized abutment. The coping and the implant abutment are then one piece, reducing the risk of gaps between the different components.

**Figure 20:**
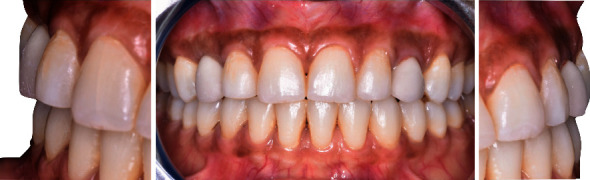
Ceramics before firing and finishing.

**Figure 21:**
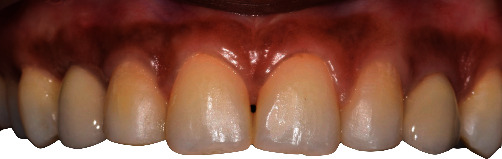
Frontal view of anterior maxilla with direct-implant fixed crowns in positions 6 and 11 showing aesthetic integration.

**Figure 22:**
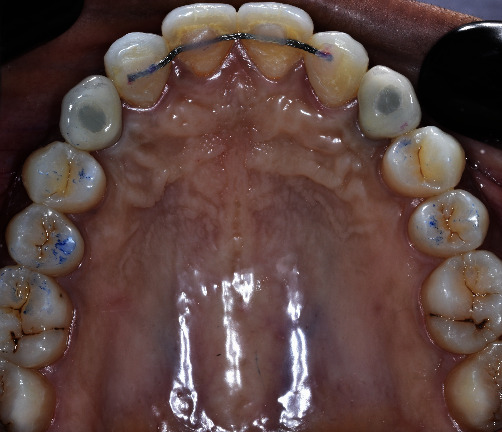
Maxilla occlusal view showing the absence of contact in static occlusion on implant fixed crowns.

The crowns were tried before finishing to check the emergence profile, interdental contact points, static and dynamic occlusions, and ceramic color (2M2 using Vita 3D-Master®, Vita) (Figure 20). When placing the direct-implant crowns, all previous points were checked, and crowns were then screwed with an insertion torque equal to 30 N cm (Figures 21 and 22).

The patient was satisfied with this rehabilitation, both from a functional and aesthetic point of view.

### 2.7. Control Visits

Controls were realized at 2 weeks and 1 month in order to check occlusion, insertion torque, plaque index, and patient comfort. All static occlusion contacts were deleted on implant fixed crowns, and canine guidance was carefully calibrated in lateral movements.

Calibrated interdental toothbrushes were prescribed in order to control plaque around implant-supported crown, especially in distal of crown in position 6.

**Figure 23:**
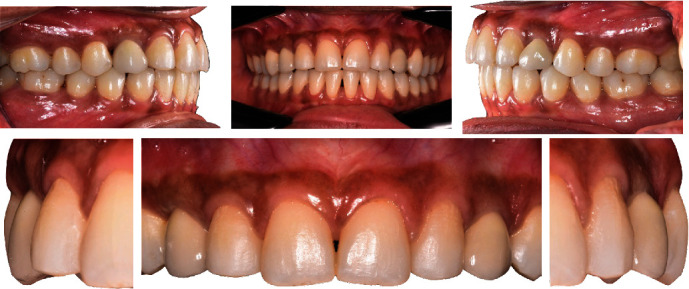
Three months follow-up showing aesthetic integration of implant fixed crowns.

A 1-year follow-up showed a total integration of crowns with profile emergence and volume apical to crowns fitting natural adjacent structures (Figure 23).

**Figure 24:**
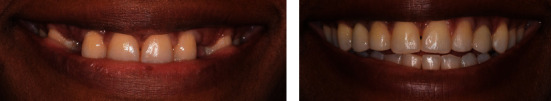
Comparison of smile results before restoration and after implant fixed rehabilitation.

Patient was completely satisfied by the aesthetic and smile results (Figure 24).

In the future, regular follow-up examinations will be carried out to ensure good tissue integration, proper plaque control, and peri-implant health.

## 3. Discussion

A long-term rehabilitation for congenital missing teeth is fundamental for patient's comfort and social life. Orthodontic treatment associated with implant fixed restoration has been described as one of the solutions for replacing lateral incisors, particularly when adjacent teeth have no filling, no color, or no size issues [13]. In this case, implants are the most conservative approach with excellent long-term results regarding function [4, 14]. Some authors recommend the use of a cantilever bonded bridge for maxillary incisor replacement. Despite the high survival rate, these rehabilitations frequently require a preparation of the abutment tooth [15–17]. With an implant rehabilitation, the adjacent teeth are not prepared and left in their current state of health and not used as part of a more extensive restoration. Moreover, prepared teeth are subject to more increased incidence of decay [18]. Various materials can be employed: metal, which produces good results but is sometimes aesthetically prejudicial, or ceramic, whose fracture is the main cause of bonded bridge failure [19]. The risk of fracture is increased when a canine is involved in the bonded bridge, due to the divergent direction of the forces applied in the propulsion and deduction movements. Narrow implants might be considered a reliable and predictable treatment from an aesthetic [20], functional, and cost-effectiveness point of view. They also could be indicated in areas in which the use of implants needs additional bone augmentation and/or expansion procedures [21].

Concerning aesthetic outcomes, especially pink aesthetic ones like gingival contours and interdental papillae, implants in anterior maxilla area are a real challenge [22]. Interdental papillae around single tooth implants depend on bone level of adjacent teeth rather than peri-implant bone level, making pink aesthetic score difficult to fully obtain [23]. In this case and to limit difficulties due to implantation in maxillary anterior area, mesialisation of canines in lateral incisor area has been realized by orthodontic treatment. Other authors have proposed also a new approach using mesialisation of canines and bicuspids and placing implants in second premolars areas to avoid anterior area implantation [24].

To avoid long-term issues, several criteria have to be analyzed before implant placement. First, evaluation of cranio-facial growth, the patient was about 30 years old with short facial type. According to Fudalej et al. [25] and Bernard et al. [8], the amount of growth is clinically insignificant for this patient allowing implant placement with a minimal risk. To prevent the infraposition implant risk, the implant was placed in a palatal position in order to facilitate the replacement or adjustment of the implant restoration in case of residual growth [26].

Second, evaluation of the future implant site. In fact, in order to place the implant in a correct 3-D position in adequation with prosthesis axis and with 1.5 mm buccal peri-implant bone, ridge augmentation is sometimes necessary. In this case, the buccolingual dimension was underestimated regarding computed tomography [27], and bone graft has been avoided using narrow implants (diameters 3.1 mm). These implant types showed no significant difference in survival rate compared to standard diameter implants [28] and are a well-documented alternative treatment. Plus, this treatment choice diminished the global treatment cost and the patient's morbidity.

Third, coronally mesiodistal dimensions have to be as needed regarding the tooth to replace. Here, two canines were restored with coronally mesiodistal dimensions of 7.5 mm, allowing the placement of small canines. These spaces would have been more opened (8 mm) during orthodontic treatment to facilitate morphological reconstruction [29].

Fourth, the apically mesiodistal dimensions are also important to protect adjacent roots. A minimum of 6 mm is needed to allow the placement of a 3.5 mm diameter implant. Here, root axes were divergent but apically mesiodistal spaces allowed the placement of a narrow implant.

Fifth, management of retention space is often necessary between orthodontic and implant treatment. In fact, healing and osseointegration take time, and temporization is necessary during this waiting time. Here, a removable solution (tray and partial denture) has been chosen and well accepted by the patient due to its affordable cost and aesthetic integration with an average smile line. A temporary resin-bonded bridge could have been more comfortable for the patient for long waiting time.

Sixth, KT height and width management must be taken into account. In fact, a lack of KT is associated with peri-implantitis, mucosal recession, and attachment loss [30]. In this case, thin KT was observed regarding implant in position 6. During the second stage of implant surgery, an augmentation of peri-implant soft tissue volume was realized using an envelope flap with a deepithelialized gingival graft [31]. This procedure was necessary for peri-implant health but also for aesthetic integration of the future implant fixed crown. The connective tissue was harvested in the crestal site to optimize aesthetic results according to pigmentations.

Finally, aesthetic integration of implant fixed crowns was managed with temporary crowns after second stage implant surgery. After soft tissue modeling, the emergence profile was impressed by adding composite resin intraorally on direct transfers before open-tray impression [32]. This technique is an easy, fast, and accurate way to transfer emergence profile but needs to be realized without delay because of soft tissue collapse after removal of the temporary crown. The use of a digital scanner to record the emergence profile is possible, but soft tissue also collapses after removal of the interim prosthesis and sulcular fluid can interfere with the recording. However, techniques are available to reduce distortion and achieve correct alignment of scans to reproduce the internal and external features of peri-implant soft tissues. A first intraoral digital impression can be done, then the provisional restoration is unscrewed, and the subgingival part of the restoration is scanned directly out of the mouth to determine its subgingival shape [33, 34]. Actually, it is also a real challenge depending on the operator and the quality of the scanner.

To optimize the final visual, teeth have been whitened before final restoration. In fact, due to irregular accumulation of chromogens and plaque around brackets, change in color of teeth can be facilitated by orthodontic treatment and needs to be treated in order to achieve complete patient satisfaction [35].

Finally, using a passive framework screwed on abutment saves from a buildup of stress at the bone/implant interface and maintains long-term osseointegration. This type of framework exhibited better passivity compared with conventionally fabricated frameworks and fabricated zirconia frameworks [36].

To resume, these types of treatment require a close partnership between all specialists and the patient. A preliminary clinical examination is essential to assess each criterion and adapt each stage of treatment to the patient's case and wishes.

## 4. Conclusion

Congenital missing lateral incisors lead to complex treatment because of the technique point of view but also because of the psychological impact on the patient.

From a technique point of view, time management is important between orthodontic treatment and implantology placement. Orthodontic goals are to open spaces and optimize aesthetic and occlusion. Implantology ones are to achieve a final functional and aesthetic rehabilitation while managing soft tissue, alveolar crest dimensions, and prosthetic integration. During all the treatment, critical steps like interim space retainer, temporary restoration before osseointegration must be managed, and patients must be informed of these ones.

From the patient point of view, this congenital affection causes aesthetic complexes since childhood and had a real psychological impact. Plus, this type of treatment is long, time-consuming, and expensive. Waiting time before final rehabilitation can last sometimes years, adding another psychological stress. A well-established treatment can help patients to better understand and appreciate all treatments.

A lot of parameters have to be analyzed like the financial aspect, the treatment length, the patient's main complaint, and expectations. Trust relationship between clinicians and the patient is the key to a successful treatment.
